# Chloroplast Acetyltransferase GNAT2 is Involved in the Organization and Dynamics of Thylakoid Structure

**DOI:** 10.1093/pcp/pcac096

**Published:** 2022-07-06

**Authors:** Marjaana Rantala, Aiste Ivanauskaite, Laura Laihonen, Sai Divya Kanna, Bettina Ughy, Paula Mulo

**Affiliations:** Molecular Plant Biology, University of Turku, BioCity A, Tykistökatu 6, Turku, FI-20520, Finland; Molecular Plant Biology, University of Turku, BioCity A, Tykistökatu 6, Turku, FI-20520, Finland; Molecular Plant Biology, University of Turku, BioCity A, Tykistökatu 6, Turku, FI-20520, Finland; Institute of Plant Biology, Biological Research Centre, Eötvös Loránd Research Network, Szeged H-6726, Hungary; Doctoral School of Biology, University of Szeged, Szeged H-6726, Hungary; Institute of Plant Biology, Biological Research Centre, Eötvös Loránd Research Network, Szeged H-6726, Hungary; Molecular Plant Biology, University of Turku, BioCity A, Tykistökatu 6, Turku, FI-20520, Finland

**Keywords:** Acetyltransferase, Arabidopsis, Light-harvesting complex, Phosphorylation, Thylakoid membrane

## Abstract

Higher plants acclimate to changes in light conditions by adjusting the thylakoid membrane ultrastructure. Additionally, excitation energy transfer between photosystem II (PSII) and photosystem I (PSI) is balanced in a process known as state transition. These modifications are mediated by reversible phosphorylation of Lhcb1 and Lhcb2 proteins in different pools of light-harvesting complex (LHCII) trimers. Our recent study demonstrated that chloroplast acetyltransferase NUCLEAR SHUTTLE INTERACTING (NSI)/GNAT2 (general control non-repressible 5 (GCN5)-related N-acetyltransferase 2) is also needed for the regulation of light harvesting, evidenced by the inability of the *gnat2* mutant to perform state transitions although there are no defects in LHCII phosphorylation. Here, we show that despite contrasting phosphorylation states of LHCII, grana packing in the *gnat2* and state transition 7 (stn7) mutants possesses similar features, as the thylakoid structure of the mutants does not respond to the shift from darkness to light, which is in striking contrast to wild type (Wt). Circular dichroism and native polyacrylamide gel electrophoresis analyses further revealed that the thylakoid protein complex organization of *gnat2* and *stn7* resembles each other, but differ from that of Wt. Also, the location of the phosphorylated Lhcb2 as well as the LHCII antenna within the thylakoid network in *gnat2* mutant is different from that of Wt. In *gnat2*, the LHCII antenna remains largely in grana stacks, where the phosphorylated Lhcb2 is found in all LHCII trimer pools, including those associated with PSII. These results indicate that in addition to phosphorylation-mediated regulation through STN7, the GNAT2 enzyme is involved in the organization and dynamics of thylakoid structure, probably through the regulation of chloroplast protein acetylation.

## Introduction

In photosynthetic light reactions, the solar energy captured by the light-harvesting complex (LHC) antenna is converted into chemical energy by the two interconnected photosystems (PSs) I and II. The LHCII-bound PSII complex feeds excited electrons to the electron transfer chain (ETC), where the electrons are passed via the cytochrome b_6_f complex and two mobile electron carriers, plastoquinone and plastocyanin, to PSI. In plants, the two PSs are spatially segregated into different thylakoid domains. PSII core (C) complex dimer together with two strongly bound (S)-LHCII trimers and two moderately bound (M)-LHCII trimers form the large C2S2M2 supercomplexes, which accommodate the appressed grana thylakoids (Caffarri et al., 2009). PSI is located in the non-appressed thylakoid stroma, which helically wind around cylindrical grana stacks (Mustárdy and Garab 2003), and grana margins, which represent an interface between grana and stroma thylakoids. A pool of loosely bound L-LHCII trimers may serve as an antenna for both PSII and PSI in grana margins (Grieco et al. 2015).

The functional balance of the ETC in constantly changing environmental conditions requires delicate regulation of light harvesting and electron transfer reactions. Rapid variations in light intensity and spectra, caused by abrupt movements of canopy or clouds in the sky, promote adjustments in the arrangement of photosynthetic proteins and the entire thylakoid macrostructure. These changes are mainly mediated by reversible phosphorylation of LHCII proteins, Lhcb1 and Lhcb2, catalyzed by the STATE TRANSITION 7 (STN7) kinase and Thylakoid-Associated Phosphatase of 38 kDa (TAP38)/Protein Phosphatase 1 (PPH1) phosphatase (Bellafiore et al. 2005, Pribil et al. 2010, Shapiguzov et al. 2010) in a process referred as state transition. Thylakoid protein complexes cover 70% of the thylakoid membrane area, and LHC complexes account for half of the protein complexes (Kirchhoff et al. 2002). Therefore, protein-phosphorylation-induced rearrangements of the LHCII proteins deploy a direct influence on the entire thylakoid macrostructure (Rantala et al. 2020). In darkness, the LHCII proteins are dephosphorylated, and the grana diameter and the number of membrane layers per grana are high (Wood et al. 2018). Exposure to low or moderate white light activates the STN7 kinase, which catalyzes the addition of a phosphate (P) group to the N-terminal threonine residues of Lhcb1 and Lhcb2 proteins. Lhcb1 phosphorylation in the PSII–LHCII supercomplexes (Galka et al. 2012, Wientjes et al. 2013) in particular induces partial destacking of grana by reducing the grana diameter and the number of membrane layers (Wood et al. 2018). The Lhcb2 phosphorylation in turn occurs in the L-LHCII trimers (Galka et al. 2012, Crepin and Caffarri 2015), which upon phosphorylation associate with the PSI complex and function as an effective antenna for PSI (Wientjes et al. 2013). It has been recently shown that LHCII phosphorylation per se, not its association with PSI, regulates grana dynamics upon light shifts. This conclusion is based on the finding showing that in contrast to *stn7*, there is no deficit in grana dynamics of the *psal* mutant, which is unable to dock phosphorylated LHCII trimer to PSI (Wood et al. 2019). The pLhcb1-mediated structural rearrangements together with the pLhcb2-mediated adjustments in light harvesting ensures sufficient excitation of the PSI complex and promote larger relative contact area between grana and stroma thylakoids, reducing the diffusion distance of the mobile electron carriers and, therefore, facilitating linear electron transfer (Joliot et al. 1992, Höhner et al. 2020, Hepworth et al. 2021).

Protein phosphorylation is among the most common and the most extensively surveyed posttranslational modification of photosynthetic proteins. However, recent analyses of Arabidopsis acetylomes have revealed numerous acetylated proteins and acetylation sites in plant chloroplasts, suggesting another important posttranslational modification involved in the regulation of chloroplast function (Bienvenut et al. 2012, Hartl et al. 2017). Notably, all protein complexes involved in the photosynthetic light reactions contain several acetylation sites (Hartl et al. 2017). Protein acetylation, which is a co- or posttranslational addition of an acetyl group from acetyl coenzyme A (Ac-CoA) to either the N-terminus of a protein or the ε-amino group of internal lysine (Lys) residues, alters the sterical and electrostatic properties of proteins by neutralizing the positive charge of the acetylation site. In N-terminal (Nt) acetylation, catalyzed by Nt-acetyltransferases (NATs), the Ac group is added to the N-terminus of a nascent protein in an irreversible manner. The reversible Lys acetylation, in turn, is catalyzed by lysine acetyltransferases (KATs) and occurs post-translationally. A recent survey on chloroplast acetyltransferases in *Arabidopsis thaliana* recognized eight chloroplast-associated acetyltransferases (GNATs) possessing dual NAT/KAT activity (Bienvenut et al. 2020).

GNAT2 (previously NSI) is the first chloroplast lysine/NAT, which has a reported role in the regulation of photosynthetic light reactions (Koskela et al. 2018). Indeed, the GNAT2 acetyltransferase is required for the regulation of light harvesting upon short-term changes in light conditions. The *gnat2* mutant is unable to form the state-transition-specific PSI–LHCII complex and balance the excitation energy between the two PSs although there are no defects in the LHCII phosphorylation (Koskela et al. 2018). The *gnat2* knockout lines (*nsi-1* and *nsi-2*) exhibit decreased Lys acetylation of several chloroplast proteins, notably PsbP-1, PsaH-1/2, LHCB1.4 and KEA1/KEA2. PsbP proteins are components of the oxygen-evolving complex, PsaH serves as a docking site for LHCII during state transitions, Lhcb1.4 is a component of the LHCII antenna, and KEA1/2 proteins function as ion transporters at the envelope membrane (Koskela et al. 2018). Further, transmission electron micrographs of the *gnat2* chloroplasts demonstrate that there is a statistically significant difference between the stacking of the *gnat2* and wild-type (Wt) grana membranes, with the *gnat2* grana stacks being more tightly packed than those of Wt (Koskela et al. 2018). These results indicate that the chloroplast acetyltransferase GNAT2 and presumably acetylation of chloroplast proteins add completely new components to the extensively studied regulatory processes; yet, the exact mechanism and the role of protein acetylation in the state transitions remain to be elucidated.

Here, we examined the light-dependent thylakoid membrane structure and dynamics and studied the distribution of the phosphorylated LHCII associated with thylakoid protein supercomplexes in the *gnat2* mutant. The results are compared to those of the *stn7* mutant, which similarly to *gnat2* is unable to form the PSI–LHCII supercomplex and balance the excitation energy transfer between the PSs. Moreover, we discuss the roles of phosphorylation and acetylation in determining the thylakoid macro-organization as well as in the induction of state transitions.

## Results

### The thylakoid grana of *gnat2* plants do not respond to the shifts from darkness to light

The dynamic remodeling of the thylakoid membrane macrostructure is mainly attributed to the changes caused by reversible LHCII phosphorylation (Kyle et al. 1983, Rozak et al. 2002, Wood et al. 2019). The tight packing of grana in darkness partially unwinds in low/moderate light, resulting in the narrowing of grana diameter (Wood et al. 2019) and in the increased area of grana margins. Sectional loosening of grana is a prerequisite for partial intermixing of the two PSs and LHCII, facilitating the contact between L-LHCII and PSI and allowing fluent electron flow between the two PSs.

**Fig. 1:**
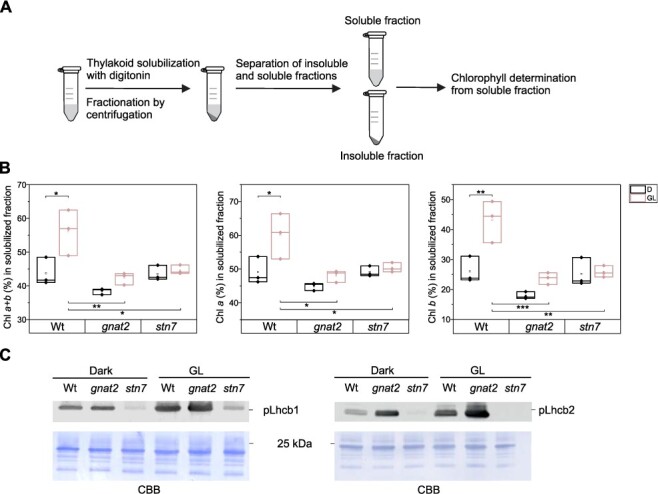
The ratio of digitonin-soluble non-appressed thylakoids (stroma lamellae and grana margins) and insoluble appressed thylakoids (grana), and the phosphorylation status of the Lhcb1 and Lhcb2 proteins upon shift from darkness to light. (A) The thylakoid membranes were fractionated into grana and stroma domain with digitonin, and (B) the ratio of the domains was estimated by measuring the amount of chlorophyll (Chl *a*, Chl *b* and Chl *a + b*) before fractionation by centrifugation (Chl 100%) and after the centrifugation from the soluble fraction. Averages and standard deviation from three biological replicates are presented. (C) The phosphorylation status of the Lhcb1 and Lhcb2 proteins was determined with specific pLhcb1 and pLhcb2 antibodies. The membranes were stained with Coomassie brilliant blue to demonstrate equal loading. Representative blots from three different biological replicates are shown. Analysis of variance (ANOVA) with Tukey test, significance * *P* < 0.05; ** *P* < 0.01; *** *P* < 0.001.

Based on electron micrographs, thylakoid membranes of *gnat2* appear more appressed than in Wt (Koskela et al. 2018). To assess whether the thylakoids of the *gnat2* mutant have the same light-dependent dynamics as Wt, the mild nonionic detergent digitonin was used to fractionate the two membrane domains. It is well established that digitonin selectively solubilizes the non-appressed thylakoid domains, leaving the grana intact (Boardman et al. 1966, Andersson and Anderson 1980, Jennings et al. 1980). Thus, the size of the solubilized fraction relative to the insoluble (grana) fraction provides indirect information on the thylakoid structure. Thylakoids isolated from Wt and the *gnat2* and *stn7* mutants after a dark period and after a subsequent light treatment were solubilized with digitonin, and the soluble and insoluble fractions were separated by centrifugation (**Fig. 1A**). The amount of digitonin-soluble, non-appressed thylakoid fraction (stroma thylakoids and grana margins) was estimated by determining the chlorophyll (Chl) concentration in the supernatant and comparing it to the Chl concentration in the sample prior to digitonin fractionation. As demonstrated in **Fig. 1B**, in Wt the proportion of Chl in the non-appressed thylakoid fraction increases significantly upon shift from darkness to light (from 44% to 56% of Chl in the solubilized fraction) concomitantly with the phosphorylation of the Lhcb1 and Lhcb2 proteins. The Chl *a/b* ratio simultaneously decreases almost 30% from 6.3 in darkness to 4.6 in light as the amount of Chl *b* in the solubilized fraction increases (**Supplementary Table S1**). Intriguingly, in the *gnat2* mutant especially the Lhcb2 proteins are highly phosphorylated already in darkness and become hyperphosphorylated upon illumination (**Fig. 1C**). Yet, the Chl proportion in the supernatant increases only marginally upon shift from darkness to light (from 38% to 42%, the shift is not statistically significant). Thus, in light the proportion of Chl in the non-appressed thylakoid fraction is significantly lower in *gnat2* as compared to Wt, demonstrating that the majority of Chl in *gnat2* is left in the insolubilized grana thylakoids regardless of the light condition and LHCII phosphorylation, which is in striking contrast to Wt (**Fig. 1B**, **C**). The Chl *a/b* ratio in *gnat2* decreases from 7.9 in darkness to 6.6 in light (17%). Despite this slight decrease, the ratio is still higher in *gnat2* in light than in Wt in darkness, and it represents the Chl *a/b* ratio of pure stroma thylakoids (Trotta et al. 2019). The solubilization data coincide with the results obtained with electron microscopy (Koskela et al. 2018): the grana are more packed in *gnat2* than in Wt and therefore it is less accessible to digitonin. Importantly, it seems that the *gnat2* thylakoid membrane lacks the ability to lose the packing dynamically upon exposure to light despite Lhcb1 and Lhcb2 (hyper)phosphorylation (**Fig. 1C**). The *stn7* mutant, which contrary to *gnat2*, shows complete dephosphorylation of LHCII proteins in all light conditions (**Fig. 1C**), behaves similarly to *gnat2*, exhibiting only marginal increase of Chl in the non-appressed thylakoid fraction upon shift from darkness to light (from 44% to 45% of Chl in the solubilized fraction) (**Fig. 1B**).

Chl *a* is bound to the PS reaction centers and the LHC antennas, whereas Chl *b* is mainly associated with the LHCII proteins. Chl *a* is a more abundant pigment in plants than Chl *b*, the ratio being typically around 3 in the thylakoids. In Wt, around half of all Chl *a* pigments but less than 30% of Chl *b* are in the non-appressed thylakoid domain in darkness (**Fig. 1B**). Upon shift to light, the amount of both Chls, but especially Chl *b*, in the solubilized fraction rises (**Fig. 1B**). This is due to the partial destacking of grana and the migration of phosphorylated LHCII toward the digitonin-accessible non-appressed thylakoid fractions. In *gnat2* and *stn7*, the proportion of both Chl *a* and Chl *b* in the non-appressed thylakoid fraction does not significantly differ from that of Wt in darkness (**Fig. 1B**). However, since the proportion of Chl *a* and *b* rises only marginally upon shift from darkness to light, the proportion of both pigments in the non-appressed region is significantly lower in *gnat2* and *stn7* as compared to Wt in light. Indeed, the proportion of Chl *b* in *gnat2* and *stn7* is roughly half of the proportion of Chl *b* in Wt after light exposure (24% in *gnat2*, 26% in *stn7* and 43% in Wt) (**Fig. 1B**). The result indicates that a large amount of LHCII in *gnat2* and *stn7* remains in the insoluble grana fraction even upon light exposure despite the opposite phosphorylation patterns of LHCII in these mutants (**Fig. 1C**).

### Macro-organization of the thylakoid membrane

**Fig. 2:**
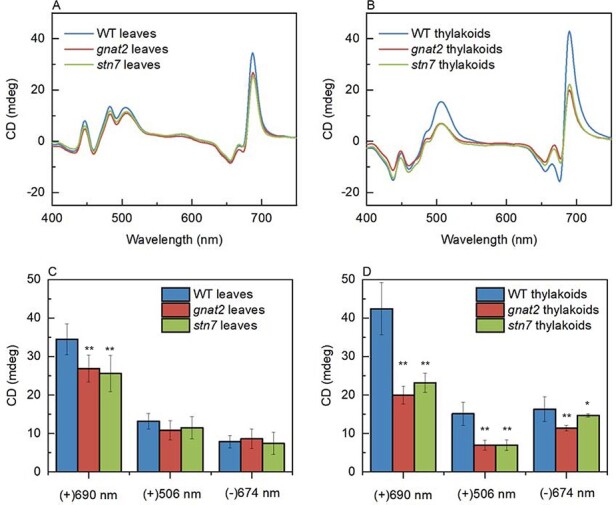
CD spectra and amplitudes of the main psi-type CD bands, (+)690 nm, (−)674 nm and (+)506 nm, of the *A. thaliana* Wt, *gnat2* and *stn7* mutant leaves (A, C) and isolated thylakoid membranes (B, D). The spectra were normalized to the red absorption maxima and represent the averages obtained from *n* ≥ 7 independent samples. Amplitudes of the psi-type CD bands were determined with a reference wavelength at 750 nm; the data represent mean ± standard error of seven biological replicates. ANOVA with Tukey test, significance * *P* < 0.05; ** *P* < 0.01.

To study the macro-organization of the thylakoid membranes of *gnat2* and *stn7* further, intact leaves and thylakoid membranes were subjected to circular dichroism (CD) spectroscopy. As shown in **Fig. 2**, the main features of the CD spectrum of Arabidopsis leaves (**Fig. 2A**) are very similar to the thylakoid membranes (**Fig. 2B**). The characteristic psi-type CD bands in Arabidopsis at (+)690 nm and (−)674 nm and (+)506 nm (Tóth et al. 2016) have been shown to originate from a long-range chiral order of the protein complexes of PSII and LHCII in the grana (Lambrev and Akhtar 2019). Additional bands at (−)650 nm in the red and at around (+)448, (−)460 and (+)484 nm in the Soret region originate from short-range excitonic interactions. The (+)690 nm (red positive) psi-type CD band of Wt leaves was significantly higher than in the *gnat2* and *stn7* leaves (**Fig. 2A**, **C**), and the same holds true for the isolated thylakoid membranes (**Fig. 2B**, **D**). These data may indicate the detachment of some LHCIIs from PSII (Kovács et al. 2006, Kiss et al. 2008, Lambrev and Akhtar 2019). The amplitude of the (−)674 nm band of mutant leaves did not show any difference compared to that of Wt leaves. However, the amplitude of (−)674 nm band in the thylakoid membranes of Wt seems to be higher than in the mutants. In the case of leaves, (+)506 nm band is similar in all the lines. However, the amplitude of the (+)506 nm band, which is generally assigned to β-carotene bound to the PSII core complexes (Tóth et al. 2016), is significantly higher in Wt compared to the *gnat2* and *stn7* mutants in the case of thylakoids. All these changes can be related to changes in the structure of LHCII and PSII–LHCII supercomplex, as it was previously shown that decreased stability of the PSII–LHCII complex, or changes in the LHCII composition and decrease in LHCII trimers, can induce the diminishment of the psi-type CD bands (Tóth et al. 2016).

### Hyperphosphorylated Lhcb2 protein in the *gnat2* mutant is associated with PSII–LHCII supercomplexes

**Fig. 3:**
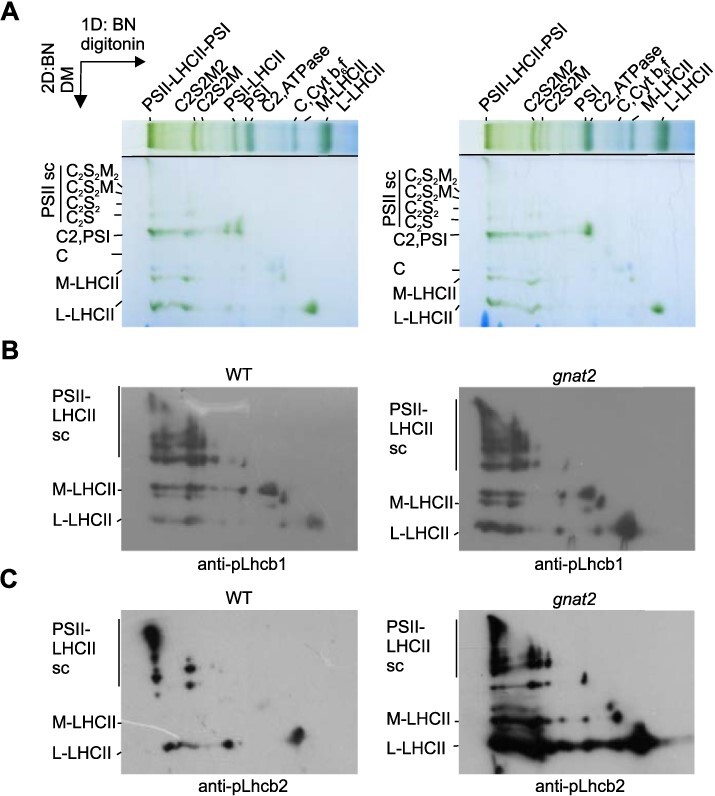
2D-BN-BN-PAGE demonstrating the distribution of pLhcb1 and pLhcb2 proteins in different pools of LHCII trimers. (A) The digitonin/ACA-solubilized protein complexes were separated by 1D-BN-PAGE (top horizontal lane). The gel strip was further solubilized with β-DM and protein complexes separated into subcomplexes by 2D-BN-BN-PAGE. The 2D gels were electroblotted on PVDF membrane and immunoprobed with (B) pLhcb1 and (C) pLhcb2 antibodies. Sc = supercomplex.

Since Lhcb2 phosphorylation, which in Wt occurs mainly in L-LHCII (Rantala et al. 2017), does not result in the formation of the PSI–LHCII complex in *gnat2* and the phosphorylated LHCII seems to be trapped in grana core, we tested to which complexes is pLhcb2 associated with in the *gnat2* mutant. We also tested the location of other major Lhcb proteins (Lhcb1-3 and pLhcb1) in *gnat2* and Wt. To address this, we solubilized the Wt and *gnat2* thylakoids with digitonin in aminocaproic acid (ACA) buffer to release labile thylakoid protein complexes from the membrane. Notably, ACA allows digitonin to access the appressed thylakoid regions, and thus the protein complexes are solubilized also from grana core (Rantala et al. 2017). The digitonin-solubilized protein complexes were separated with blue-native polyacrylamide gel electrophoresis (BN-PAGE) and the lanes were cut and further solubilized with a slightly stronger detergent, β-dodecyl-maltoside (DM), which destroys weak hydrophobic interactions between labile protein complexes (Wittig et al. 2006). The DM-solubilized lanes were separated on a sequential BN-PAGE to release smaller subcomplexes. In **Fig. 3A**, the protein complexes below the diagonal represent the subcomplexes released from the digitonin-solubilized complexes. DM mainly disconnects LHCII from the PSs, releasing all the loosely bound L-LHCII from the PSs and partially M-LHCII from the C2S2M2 and C2S2M supercomplexes. To assess in which pools of LHCII (S-LHCII/M-LHCII/L-LHCII) Lhcb1 and Lhcb2 phosphorylation take place, the 2D-BN-BN-gels were electroblotted and probed with pLhcb1- and pLhcb2-specific antibodies. In Wt and *gnat2*, pLhcb1 is found associated with the PSII–LHCII supercomplexes and detached M- and L-LHCII (**Fig. 3B**, left panel). In Wt, the pLhcb2 protein is mainly found in the L-LHCII (**Fig. 3C**, left panel), which is in line with previous observations (Crepin and Caffarri 2015, Rantala et al. 2017). However, some pLhcb2 is also associated with the PSII–LHCII–PSI complex, which contains L-LHCII (**Fig. 3C**, left panel). Intriguingly, in *gnat2* the Lhcb2 protein is hyperphosphorylated and not only associated with the L-LHCII trimers, but also found abundant in the PSII supercomplexes and in M-LHCII disconnected from the PSII–LHCII supercomplexes (**Fig. 3C**, right panel). Probing the 2D-BN with Lhcb2-specific antibody confirms the presence on Lhcb2 protein in the M-LHCII in the *gnat2* mutant (**Supplementary Fig. S1B**). No differences were observed in the distribution of pLhcb1, Lhcb1 and Lhcb3 proteins in the *gnat2* mutant as compared to Wt (**Fig. 3B**, **Supplementary Fig. S1A, C**).

### Detection of PSI–LHCII in the Mg^2+^-depleted randomized thylakoids of the *gnat2* mutant

In light, Lhcb1 and Lhcb2 proteins become phosphorylated and the thylakoid grana partially unwind. The partial unwinding of grana and Lhcb2 phosphorylation in the L-LHCII trimers increase the affinity of L-LHCII trimers toward PSI, allowing the formation of the PSI–LHCII complex and resulting in increased direction of excitation energy toward PSI. To uncover whether the inability to form the PSI–LHCII complex in *gnat2* is due to the restricted mobility of phosphorylated Lhcb proteins from the tightly appressed grana to the PSI-enriched stroma fraction and grana margins, the thylakoids were isolated with and without MgCl_2_. As cation depletion (most importantly Mg^2+^) is known to result in the destacking of grana and randomization of thylakoid membrane complexes (Barber, 1980, Murakami and Packer 1971), thylakoid isolation without MgCl_2_ should allow the interaction between pLhcb2 and PSI if the structural rigidity of the membrane alone limits the interaction of PSI and LHCII.

**Fig. 4:**
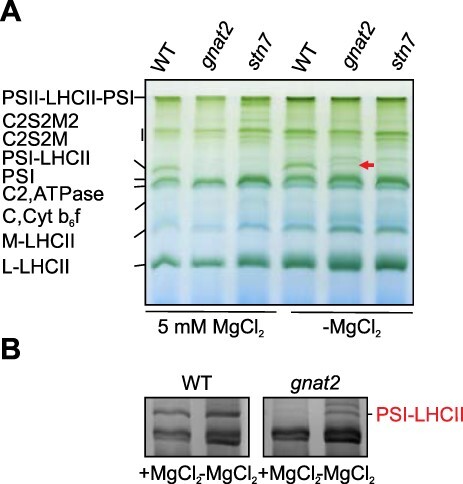
Accumulation of PSI–LHCII complex. (A) Protein complexes from the Wt, *gnat2* and *stn7* thylakoids (±Mg^2+^) solubilized with digitonin were separated with BN-PAGE. (B) Coomassie staining was used for better visualization of the PSI–LHCII accumulation in *gnat2.*

Protein complexes were solubilized from the membrane with digitonin and subsequently separated by BN-PAGE. In line with previous results, the PSI–LHCII supercomplex was completely absent in the *gnat2* and *stn7* mutants as compared to Wt when thylakoids were isolated with buffers containing MgCl_2_ (**Fig. 4**). However, when thylakoids were isolated without Mg^2+^ to obtain randomized thylakoid membranes, a small amount of the PSI–LHCII complex appeared in the *gnat2* mutant (**Fig. 4**, **Supplementary Fig. S2**). For better visibility, the BN gel was dyed with Coomassie stain (**Fig. 4B**). Although a small amount of PSI–LHCII accumulated in *gnat2* in the Mg^2+^-depleted thylakoids, it is clear that artificial destacking of grana did not restore the capacity to normally accumulate the PSI–LHCII complex.

## Discussion

The intensity and spectral properties of light are changing seasonally and according to the diurnal cycle. Further, short-term changes in the light condition occur all the time as the clouds pass by and the canopy overshadows the undergrowth. The photosynthetic machinery needs to react to these changes in order to maximize light utilization and to avoid destructive effects of excess light. Reversible LHCII phosphorylation is essential and among the most intensively scrutinized photosynthetic regulatory mechanisms, which ensures balanced excitation energy distribution and fluent electron flow between the two spatially segregated PSs in the plant thylakoid membrane. The molecular mechanisms behind LHCII phosphorylation and its role in the regulation of grana dynamics have been characterized in detail (Rochaix 2007, Tikkanen et al. 2008, Johnson and Wientjes 2020). Yet, the recent discovery of chloroplast acetyltransferase GNAT2, whose mutation can block the state transitions (Koskela et al. 2018), has brought new complexity to the regulation of the light-harvesting system. Recent proteomic surveys have revealed that several proteins involved in photosynthetic light reactions are heavily acetylated (Hartl et al. 2017, Uhrig et al. 2019), but so far GNAT2 is the first chloroplast lysine/Nt acetyltransferase, which has a reported role in the regulation of photosynthetic light reactions (Koskela et al. 2018). In the *gnat2* mutant, the phosphorylated LHCII trimer is unable to dock to the PSI complex and thus to direct excitation energy toward PSI (Koskela et al. 2018). Here we further characterized LHCII phosphorylation and thylakoid membrane dynamics in the *gnat2* mutant. As a control we used the *stn7* mutant, which alike to *gnat2* is unable to perform state transitions; however, unlike *gnat2*, it shows the complete dephosphorylation of LHCII proteins due to the absence of the LHCII kinase.

The thylakoid membrane of land plants is characterized by the formation of appressed grana stacks, which are surrounded by helically wound stroma lamellae. Grana-located PSII–LHCII complexes are segregated from the PSI complexes, which due to their stromal protrusion cannot fit in the tightly appressed grana stacks. Strict lateral heterogeneity of the PSs is true only in darkness, when LHCII is dephosphorylated. LHCII phosphorylation upon low or moderate white light reduces the grana diameter and the number of membrane layers per grana, allowing the formation of the PSI–(L)-LHCII complex (Wood et al. 2019). The lateral reorganization of the photosynthetic machinery and the consequent rearrangement of the entire thylakoid membrane allow optimized light harvesting for both PSs and ensure fluent electron flow between the PSs.

Electron micrographs demonstrated that the *gnat2* mutant has slightly more packed grana thylakoids than Wt (Koskela et al. 2018). Here, we used the digitonin fractionation of the thylakoid membrane into grana and stroma lamellae and subsequent spectroscopic analysis of pigment composition of the two fractions to estimate the ratio of digitonin-soluble stroma lamellae to insoluble grana membranes. The pigment composition of the plant thylakoid fractions was determined following darkness and subsequent exposure to moderate white light in order to examine the dynamics of the thylakoid membrane. In Wt, the amount of chlorophyll (especially LHCII-bound Chl *b*) in the non-appressed thylakoid fraction (stroma lamellae and grana margins) increased markedly upon shift from darkness to light (**Fig. 1B**), while the Lhcb1 and Lhcb2 phosphorylation was simultaneously induced (**Fig. 1C**). These observations are in line with previous reports (Wood et al. 2018, 2019, Johnson and Wientjes 2020): in darkness, the thylakoid membrane is highly appressed and the majority of Chl is in the grana stacks. Light exposure induces LHCII phosphorylation, which is followed by the loosening of grana and migration of LHCII toward the non-appressed thylakoid domains, increasing the amount of the digitonin-soluble membrane fraction. In the *gnat2* and *stn7* mutants, the Chl concentration in the non-appressed thylakoid membrane increases only marginally upon shift from darkness to light despite the opposite phosphorylation patterns of LHCII and, therefore, upon illumination remains at a significantly lower level as compared to Wt (**Fig. 1B**, **C**). Notably, it is particularly the amount of LHCII-bound Chl *b*, which is significantly lower in the non-appressed thylakoid fraction in the *gnat2* and *stn7* mutants as compared to Wt (**Fig. 1B**). Thus, it seems that despite the high phosphorylation of LHCII proteins (**Fig. 1C**), the thylakoid membrane remains highly appressed in the *gnat2* mutant and a large amount of LHCII remains trapped in grana thylakoids regardless of the light condition. Wood et al. (2019) previously showed that the *psal* mutant, which is incapable of forming the PSI–LHCII complex (state transition complex), is able to dynamically remodel the thylakoid membrane. Since the *psal* mutant is able to phosphorylate LHCII, it was suggested that LHCII phosphorylation, not the accumulation of the PSI–LHCII complex, regulates grana dynamics. Interestingly, the *gnat2* mutant, similarly to *psal* mutant, does not accumulate PSI–LHCII complex despite LHCII phosphorylation, but unlike *psal* has lost the ability for dynamic thylakoid remodeling. These results suggest that although LHCII phosphorylation undoubtedly is the main regulator of the grana dynamics, also other factors, possibly protein acetylation, are required for the regulation.

Since phosphorylated L-LHCII is unable to associate with the PSI complex in the *gnat2* mutant (Koskela et al. 2018) and a large amount of LHCII remains in grana despite LHCII phosphorylation (**Fig. 1B**, **C**), we determined in which pools of LHCII the Lhcb2 phosphorylation takes place. Previously, it has been shown that the Lhcb1 phosphorylation occurs particularly in the S-LHCII trimers associated with PSII–LHCII supercomplexes, whereas the Lhcb2 phosphorylation takes place almost exclusively in the pool of L-LHCII, where it also orchestrates its association with the PSI complex (Galka et al. 2012, Wientjes et al. 2013, Crepin and Caffarri 2015). Here we used a 2D-BN-BN system to separate the sub-populations of LHCII complexes (S/M and L) and a specific pLhcb1 and pLhb2 as well as Lhcb1-3 antibodies to locate the phosphorylated Lhcb proteins in different LHCII trimers. Intriguingly, in the *gnat2* mutant, the phosphorylated Lhcb2 protein was found in L-LHCII, disconnected M-LHCII and LHCII trimers associated with the PSII–LHCII supercomplexes, while in Wt pLhcb2 was found predominantly in the pool of L-LHCII as it has been shown before (**Fig. 3**; Galka et al. 2012, Crepin and Caffarri 2015, Rantala et al. 2017). We have previously shown that the overall abundance of Lhcb2 in *gnat2* has not markedly changed (Koskela et al. 2018) and that the composition of L-LHCII is not altered in *gnat2* and *stn7* as compared to Wt (Koskela et al. 2020). Since the L-LHCII trimers are involved in the state transitions, it is intriguing that most striking differences between the *gnat2* mutant and Wt plants were in the presence of pLhcb2 in the detached M-LHCII and LHCII trimers associated with PSII, not in the L-LHCII trimers (**Fig. 3C**). In Arabidopsis, the M-LHCII trimer is normally enriched with the Lhcb1 and Lhcb3 proteins, while Lhcb2 is rarely present (Galka et al. 2012). These results were supported by CD spectroscopy, which showed a similar macro-organization of pigment protein complexes (especially LHCII and PSII–LHCII) in the *stn7* and *gnat2* mutants (**Fig. 2**), despite the drastic differences in the phosphorylation of LHCII.

Finally, we artificially destacked the thylakoid membranes to assess whether only the structural inflexibility of the thylakoid membrane prevents the contact between L-LHCII and PSI. As discussed above, the LHCII phosphorylation is normally associated with partial destacking of grana (e.g. Kyle et al. 1983, Wood et al. 2019). Since in artificially destacked thylakoids of *gnat2* only a negligible amount of the PSI–LHCII complex was formed (**Fig. 4**), it appears that the inability to destack the grana is not the primary cause for the defective formation of the PSI–LHCII complex in the *gnat2* mutant. Further research is still needed to assess the ultimate reason for the inability to accumulate the PSI–LHCII complex in the *gnat2* mutant.

## Materials and Methods

### Plant material, light treatments and thylakoid membrane isolation

*Arabidopsis thaliana* ecotype Columbia-0 (Wt) and the *gnat2*(*nsi-1*, SALK_033944; Koskela et al. 2018) and *stn7* mutants (SALK_073254) were grown under a photon flux density of 120 µmol m^−2^ s^−1^ in an 8 h light/16 h dark regime at 23°C and in a relative humidity of 50%. Osram Powerstar HQI-BT 400 W/D PRO Daylight served as a light source. Five-week-old plants were kept in darkness for 16 h and subsequently shifted to light 120 µmol photons m^−2^ s^−1^ for 3 h. Six fresh rosettes were collected in 80 ml of ice-cold grinding buffer [50 mM 4-(2-hydroxyethyl)-1-piperazineethanesulfonic acid (HEPES)/KOH (pH 7.5), 330 mM sorbitol, 5 mM MgCl_2_, 5 mM sodium-L-ascorbate, 0.1% bovine serum albumin (BSA) and 10 mM sodium fluoride]. The suspension was filtered through Miracloth followed by centrifugation at 2,739×*g* at 4°C for 5 min. The pellet was resuspended in 15 ml of shock buffer [50 mM HEPES/KOH (pH 7.5), 5 mM MgCl_2_ and 10 mM sodium fluoride] and centrifuged at 2,739×*g* at 4°C for 5 min. The pellet was washed with 15 ml of storage buffer [50 mM HEPES/KOH (pH 7.5), 100 mM sorbitol, 5 mM MgCl_2_ and 10 mM sodium fluoride], followed by centrifugation at 2,739×*g* at 4°C for 5 min. Finally, the pellet was resuspended in storage buffer. To obtain thylakoids with destacked grana, the thylakoids were isolated without MgCl_2_ in the buffers (for other isolations 5 mM MgCl_2_ was supplemented in all isolation buffers). Chlorophyll concentration was determined from the samples according to Porra et al. (1989).

### Thylakoid sub-fractionation and chlorophyll determination from the supernatant

The isolated thylakoid membranes were fractionated into grana and stroma fractions with digitonin. The thylakoid samples were diluted in BTH buffer [25 mM Bis/Tris/HCl (pH 7.0), 20% (w/v) glycerol, 0.25 mg/ml Pefabloc and 10 mM NaF] to a concentration of 1 mg/ml. An equal volume of 2% digitonin (Merck Life Sciences, Keilaranta 6, Espoo, Finland) in BTH buffer was added to the sample to achieve the final concentration of 0.5 mg/ml of Chl and 1% digitonin in the sample. The sample was solubilized for 8 min at room temperature (RT) in continuous mixing. The insoluble (grana) and soluble (stroma thylakoids) fractions were separated by centrifugation at 18,000×*g* for 20 min, at 4°C. After centrifugation, 10 µl of supernatant was added to 990 µl of buffered acetone (80% acetone and 2.5 mM HEPES/KOH pH 7.6), and the Chl concentration and Chl *a/b* ratio were determined by measuring the absorbance at wavelengths of 663.6, 646.6 and 750 nm. Chl *a* and *b* concentrations were calculated using the following formulas—Chl *a*: (1/10)*[12.25*(A664-A750) − 2.55*(A646-A750)]; Chl *b*: (1/10)*[20.31*(A646-A750) − 4.91*(A664-A750)] according to Porra et al. (1989).

### Circular dichroism measurements

CD spectra from control (Wt) and mutant (*stn7* and *gnat2*) leaves and isolated thylakoids of *A. thaliana* were recorded at room temperature in the spectral range of 400–800 nm with 3 nm spectral resolution using a J815 spectropolarimeter (JASCO Europe srl, Via L. Cadorna 1, Cremella, Italy). The scan speed was set to 100 nm min^−1^ with an integration time of 2 s. The Chl concentration of the thylakoid membranes was adjusted to 20 μg ml^−1^, and the spectra were measured in a cuvette with a 1 cm optical path length. Intact leaves supported by a flat cell were placed perpendicularly to the optical path. For baseline correction, an empty cell was used for leaves; a resuspension buffer was used for thylakoids. Multiple, independent CD scans were recorded and averaged. CD spectra were normalized to the absorption maxima (680 nm) with a reference wavelength at 750 nm. Amplitudes of (+)690 nm and (−)674 nm and (+)506 nm psi-type CD bands were calculated to compare the difference between psi-type CD in the red and blue spectral regions of the leaves and thylakoids.

### SDS-PAGE

The isolated thylakoids were solubilized with a solubilization buffer [138 mM Tris/HCl pH 6.8; 6 M urea, 22.2% glycerol (v/v) and 4.4% sodium dodecyl sulfate (SDS)] containing 10% β-mercaptoethanol, and 0.5 µg of Chl was loaded on SDS-PAGE (12% w/v polyacrylamide and 6 M urea) and separated under a constant current of 10 mA. The proteins were electroblotted to polyvinylidene difluoride (PVDF) membranes and immunoprobed with pLhcb1 and pLhcb2 antibodies (Agrisera AS13 2704 and AS13 2705, 1:10 000 dilution in 1% BSA).

### Blue-native PAGE

To solubilize the thylakoid protein complexes from the thylakoid samples isolated with or without 5 mM MgCl_2_, the samples were diluted in BTH buffer [25 mM Bis/Tris/HCl (pH 7.0), 20% (w/v) glycerol, 0.25 mg/ml Pefabloc and 10 mM NaF]. An equal volume of 2% digitonin (Merck, Calbiochem) in BTH buffer was added to the sample to achieve a final concentration of 0.5 mg ml^−1^ of Chl and 1% digitonin in the sample and separated with BN-PAGE as described previously (Rantala et al. 2018b). To investigate the distribution of Lhcb2 proteins in different pools of LHCII trimers, 2D-BN-BN-PAGE was performed as described in (Rantala et al. 2018a). The 2D-BN gels were electroblotted to PVDF membranes and immunoprobed with pLhcb1 and pLhcb2 antibodies (Agrisera AS13 2704 and AS13 2705, 1:10,000 dilution in 1% BSA) and with Lhcb1, Lhcb2 and Lhcb3 antibodies (Agrisera AS01 004, AS01 003 and AS01 002). (1:2,000–1:5,000 dilution in 1% BSA).

## Supplementary Material

pcac096_Supp

## Data Availability

The data underlying this article are available in the article and in its online supplementary material. **Accession Numbers:** AT1G32070 (GNAT2); AT1G68830 (STN7); *gnat2* (SALK_033944); *stn7* (SALK_073254).
