# Supplementary material for: Chloroplast Acetyltransferase GNAT2 is Involved in the Organization and Dynamics of Thylakoid Structure
Source: Plant Cell Physiol. 2022 Jul 6;63(9):1205–14. doi: 10.1093/pcp/pcac096 (PMC9474947; doi:10.1093/pcp/pcac096)
Supplement: pcac096_Supp [file pcac096_supp.zip › pcp-2022-e-00162-File006.pdf]

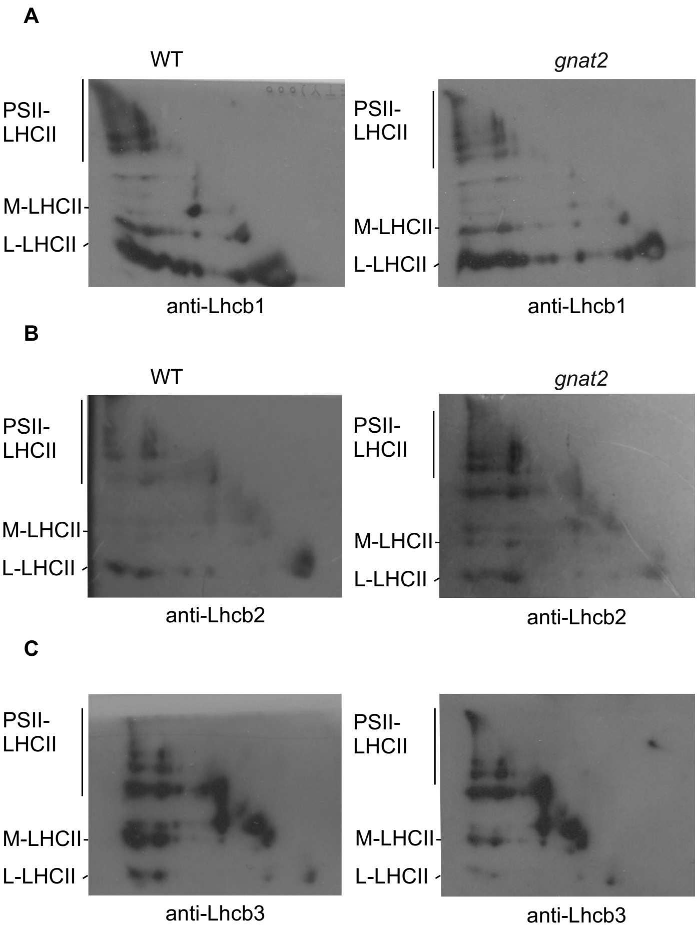

Figure S1. 2D-BN-BN-PAGE demonstrating the distribution of Lhcb1-3 proteins in different pools of LHCII trimers. The digitonin solubilized protein complexes from Wt and *gnat2* thylakoids were separated by 2D-BN-BN-PAGE. The 2D gels were electroblotted oon PVDF membrane and immunoprobed with (A) Lhcb1, (B) Lhcb2 and (c) Lhcb3 antibodies.

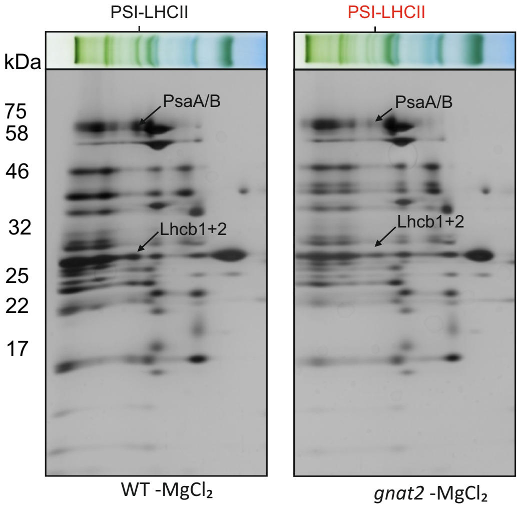

Figure S2. Representative 2D-BN-SDS gels of Wt and gnat2 thylakoids (-MgCl2) showing the accumulation of PSI-LHCII complex. Protein complexes from Wt and *gnat2* thylakoids (- Mg2+) solubilized with digitonin were first separated with BN-PAGE and then by SDS-PAGE. Proteins were visualized using SYPRO® Ruby staining according to Invitrogen Molecular Probes™ instructions.

	Dark<br>chl a/b	Light<br>chl a/b
**Wt**	6,3 ± 0,46	4,6 ± 0,27
gnat2	7,91 ± 0,81	6,59 ± 0,37
stn7	6,5 ± 1,05	6,41 ± 0,49

Table S1. Chl a/b ratio in the soluble fraction after digitonin fractionation. Mature plants were kept 16h in darkness and subsequently exposed to light for two hours. Thylakoid membranes were fractionated into grana and stroma domain with digitonin and the chlorophyll a/b ratio was determined from the supernatant. Three biological replicates were measured and averages and standard deviations are presented.
